# Supplementary material for: Personalized Medicine in Parkinson’s Disease: New Options for Advanced Treatments
Source: J Pers Med. 2021 Jul 10;11(7):650. doi: 10.3390/jpm11070650 (PMC8303729; doi:10.3390/jpm11070650)
Supplement: Supplementary file 1 [file jpm-11-00650-s001.zip › Personalized Medicine in PD Table S1.pdf]

Table S1. Comparison of DBS, MRgFUS, Radiofrequency, and Gamma Knife

	**DBS**	**MRgFUS**	**Radiofrequency**	**Gamma knife**
Tissue damage	Minimum	+	+	+
Reversibility	+	-	-	-
Adjustable	+	-	-	-
Anesthesia	General (IPG implantation)	Local	Local	Local
Time to effect	Immediate	Immediate	Immediate	One month
Burr hole craniotomy	+	-	+	-

DBS: deep brain stimulation; MRgFUS: MR-guided focused ultrasound; IPG: Implantable Pulse Generator.
