# Supplementary material for: Personalized Medicine in Parkinson’s Disease: New Options for Advanced Treatments
Source: J Pers Med. 2021 Jul 10;11(7):650. doi: 10.3390/jpm11070650 (PMC8303729; doi:10.3390/jpm11070650)
Supplement: Supplementary file 1 [file jpm-11-00650-s001.zip › Personalized Medicine in PD Table S2.pdf]

Table S2. Effect of DBS on individual symptoms for each target

	STN	GPi	Thalamus (Vim)	PPN
Tremor	+	+	++	-
Bradykinesia	++	+	±	±
Rigidity	++	++	+	-
Postural instability	+	-	-	+
Wearing-off	++	++	-	-
Dyskinesia	+	++	-	-
Freezing of gait	±	-	-	+
Gait disturbance	-	-	-	+

++: Extremely effective; +: Effective; ±: Slightly effective; -: No effect.

DBS: Deep brain stimulation; STN: subthalamic nucleus; GPi: globus pallidus internus; Vim: ventrointermedius;

PPN: pedunculopontine nucleus.
