# Supplementary material for: Personalized Medicine in Parkinson’s Disease: New Options for Advanced Treatments
Source: J Pers Med. 2021 Jul 10;11(7):650. doi: 10.3390/jpm11070650 (PMC8303729; doi:10.3390/jpm11070650)
Supplement: Supplementary file 1 [file jpm-11-00650-s001.zip › Personalized Medicine in PD Table S3.pdf]

Table S3. Comparison of DBS and LCIG

	DBS	LCIG
Age >70 year	△	○
Age ≦70 year	○	○
Moderate psychiatric symptoms due to illness	×	○
Moderate psychiatric symptoms due to drugs	△	○
Severe psychiatric symptoms	×	△
Moderate cognitive dysfunction	×	○
No caregiver	△	×

○: Indication; △: Attention; ×: Ineligible.

DBS: Deep brain stimulation; LCIG: Levodopa-carbidopa intestinal gel.
