# Supplementary material for: Personalized Medicine in Parkinson’s Disease: New Options for Advanced Treatments
Source: J Pers Med. 2021 Jul 10;11(7):650. doi: 10.3390/jpm11070650 (PMC8303729; doi:10.3390/jpm11070650)
Supplement: Supplementary file 1 [file jpm-11-00650-s001.zip › Personalized Medicine in PD Table S4.pdf]

Table S4. A list of concerns to check when considering DBS

	**Major problem**	**Minor problem**
Neurologist	Diagnostic certainty, Response to L-dopa, High-risk medical complications of surgery (cardiac disease, hypertension, diabetes, nutritional status, obesity, respiratory disease, renal disease, liver disease, abnormal blood coagulation)	Age, Freezing gait, Severe postural instability, Low-risk medical complications of surgery
Neurosurgeon	High-risk of surgical complications	Brain atrophy, Past history of brain surgery and stroke
Psychiatrist	Dementia, Severe depression, ICD, Psychiatric symptoms	Mild cognitive impairment, Depression, Anxiety, Apathy, DDS
Therapist	High risk of falling, High dependence on ADL	Freezing of gait, Severe postural instability, Abnormal posture of the trunk
Nurse		No caregivers, Lack of therapeutic goals, Gap between expectation and reality
Dentist		Dysphagia, Aspiration risk

DBS: deep brain stimulation; ICD: impulse control disorder; DDS: dopamine dysregulation syn-drome; ADL: activities of daily living.
