# Supplementary material for: Personalized Medicine in Parkinson’s Disease: New Options for Advanced Treatments
Source: J Pers Med. 2021 Jul 10;11(7):650. doi: 10.3390/jpm11070650 (PMC8303729; doi:10.3390/jpm11070650)
Supplement: Supplementary file 1 [file jpm-11-00650-s001.zip › Personalized Medicine in PD Table S5.pdf]

Table S5. A list of concerns to check when considering LCIG

	Major problem	Minor problem
Neurologist	Diagnostic certainty, Response to L-dopa, High-risk medical complications of surgery (cardiac disease, hypertension, diabetes, nutritional status, obesity, respiratory disease, renal disease, liver disease, abnormal blood coagulation)	Age, Low-risk medical complications of surgery
Gastroenterologist Colorectal surgeon	Removed stomach, Abdominal organ position variation, Risk of surgical complications	Past history of abdominal Surgery, Gallstones
Psychiatrist	Severe dementia, Severe psychiatric symptoms	Dementia, Depression, Anxiety, Apathy, ICD, DDS
Therapist	High risk of falling, High dependence on ADL	Freezing of gait, Severe postural instability, Abnormal posture of the trunk
Nurse	No caregivers	Uncooperative caregivers, Lack of therapeutic goals, Gap between expectation and reality
Dentist		Dysphagia, Aspiration risk

LCIG: Levodopa-carbidopa intestinal gel; ICD: impulse control disorder; DDS: dopamine dysregulation syndrome; ADL: activities of daily living.
